# Early risk detection of metabolic syndrome using sex-specific machine learning models in military personnel

**DOI:** 10.3389/fpubh.2025.1625461

**Published:** 2025-10-31

**Authors:** Wei-Yun Wang, Yi-Syuan Wu, Yen Huang, Wen-Chii Tzeng

**Affiliations:** ^1^Department of Nursing, Tri-Service General Hospital, National Defense Medical University, Taipei, Taiwan; ^2^College of Nursing, National Defense Medical University, Taipei, Taiwan; ^3^Department of Computer Science and Information Engineering, National Taitung University, Taitung, Taiwan; ^4^Department of Nursing, Tri-Service General Hospital Songshan Branch, Taipei, Taiwan

**Keywords:** metabolic syndrome, machine learning, predictive model, sex differences, military personnel, precision prevention

## Abstract

Metabolic syndrome is a critical predictor of future cardiometabolic disease and an emerging public health concern, particularly in high-demand populations such as military personnel. This study aimed to develop and evaluate sex-specific machine learning models for the early detection of metabolic syndrome using annual health check data. We analyzed records from 179,620 Taiwanese Air Force personnel between 2014 and 2022, incorporating demographic, anthropometric, clinical, lifestyle, mental health, and biochemical variables. Six machine learning algorithms—including logistic regression, random forest, K-nearest neighbor, support vector machine, neural network, and naïve Bayes—were trained separately for men and women. Among these models, logistic regression outperformed the others, achieving an accuracy and area under the curve (AUC) of 0.89. Body mass index, age, and alanine aminotransferase levels were consistent predictors across sexes. For men, total cholesterol and uric acid contributed significantly, while hemoglobin and hematocrit were more predictive in women. These findings demonstrate that sex-specific predictive models can support early identification of individuals at high risk for metabolic syndrome, enabling targeted prevention strategies and strengthening population health efforts in military populations and other young to middle-aged adult groups.

## 1 Introduction

Metabolic syndrome (MetS) is a cluster of interrelated conditions including obesity, elevated blood glucose, dyslipidemia, and hypertension. These conditions frequently co-occur in individuals at increased risk of cardiovascular disease and type 2 diabetes and are strong predictors of morbidity and mortality (1). As of 2018, the global prevalence of MetS was estimated at 25% and has since continued to rise, making it a growing public health concern (2). Among Air Force personnel, high stress levels increase susceptibility to metabolic disorders (3), underscoring the need for early risk detection and targeted prevention strategies in this population.

Numerous factors contribute to MetS, including age, sex (3, 4), chronic disease history (5), family history (5), and behaviors such as smoking (6), alcohol consumption (7), betel nut use (5), and physical inactivity (8). Betel nut chewing is particularly relevant in Asian populations, where its high prevalence and established associations with central obesity, dyslipidemia, and impaired glucose regulation make it a culturally specific conditioning factor in the development of MetS (9). Although body mass index (BMI) is frequently used in population screening to identify individuals at risk (10), it is not itself a causal determinant of MetS. Instead, the pathophysiological link between MetS and cardiometabolic disease arises from the quantity, distribution, and functionality of adipose tissue, with visceral fat and dysfunctional adipose compartments playing a more critical role than overall body weight (11). This distinction underscores the need to interpret BMI cautiously and to consider more direct indicators of body fat and adiposity function in risk assessments. Mental health status (12) and biochemical indicators—such as white blood cell count, hemoglobin, total cholesterol, alanine aminotransferase (ALT), and uric acid level (13, 14)—also show strong associations with MetS risk. While many predictive models have been developed, most rely on traditional statistical methods such as logistic regression and may not fully capture complex interactions among variables.

Machine learning offers a data-driven alternative capable of analyzing high-dimensional health data and identifying nonlinear patterns (15). It has shown promise in disease prediction across various health domains. This study focuses on six widely used machine learning algorithms: logistic regression (LR), random forest (RF), K-nearest neighbor (KNN), support vector machine (SVM), neural network (NN), and naïve Bayes (NB) (16, 17). Each algorithm varies in structure and learning mechanism, providing different strengths in predictive modeling (18–20).

Despite increasing interest in machine learning for health prediction (21), its application to MetS risk detection in military populations remains underexplored. This study aims to evaluate the predictive accuracy of multiple machine learning models for MetS using annual health check data from Taiwanese Air Force personnel. We further examine how sex-specific models may enhance prediction by identifying distinct risk profiles in men and women. Our goal is to inform early detection and personalized prevention strategies, contributing to improved metabolic health and operational readiness in high-demand populations.

## 2 Materials and methods

### 2.1 Study design and cohort

This population-based study used data from the Taiwanese Military Health Management Information System, which collects annual worksite health examination data from active-duty personnel. We included Air Force members aged 18 to 58 years who underwent health screenings between 2014 and 2022. Data included demographic characteristics, anthropometric measures, medical history, lifestyle behaviors, mental health indicators, and biochemical parameters. All procedures followed the ethical standards of the 1975 Declaration of Helsinki and were approved by the Institutional Review Board of Tri-Service General Hospital, Taiwan (approval number: A202305142).

### 2.2 Measures

We initially examined 36 features potentially associated with MetS (22), grouped into three categories: (1) demographic and anthropometric; (2) clinical, lifestyle, and mental health; and (3) biochemical.

#### 2.2.1 Step 1: demographic and anthropometric features

Age, sex, waist circumference, and BMI were included. BMI was calculated as weight (kg) divided by height squared (m^2^).

#### 2.2.2 Step 2: clinical, lifestyle, and mental health features

Clinical features included history of chronic disease, family history, and blood pressure (systolic and diastolic). Lifestyle features included smoking, betel nut use, alcohol consumption, physical activity, rapid fatigue during exercise, infection within 1 month, and regular medication use. The mental health features—insomnia, depression, hostility, anxiety, interpersonal sensitivity, and suicidal ideation—were assessed using the Brief Symptom Rating Scale-5 (BSRS-5), a validated five-item scale scored on a 5-point Likert scale (0–4) (23, 24). Higher scores indicated poorer psychological well-being. The Cronbach’s *α* value for the BSRS-5 ranges from 0.77 to 0.90 (23).

#### 2.2.3 Step 3: biochemical features

Biochemical features included liver function markers—aspartate aminotransferase (AST) and alanine aminotransferase (ALT); renal function markers—blood urea nitrogen (BUN) and creatinine; hematological parameters—red blood cell (RBC) count, white blood cell (WBC) count, hemoglobin, hematocrit, and platelet count; and cardiovascular indicators—total cholesterol (TC), uric acid (UA), triglycerides, high-density lipoprotein cholesterol level (HDL-C), low-density lipoprotein cholesterol (LDL-C), and fasting plasma glucose. The Air Force personnel fasted overnight before venous blood collection. Samples were processed using a clinical chemistry analyzer (ADVIA1800, Siemens, United States).

### 2.3 Study outcomes

The study outcome was the occurrence of MetS during a 9-year surveillance period. MetS diagnosis followed the modified National Cholesterol Education Program Adult Treatment Panel III criteria, with modifications from the International Diabetes Federation, which accounts for waist circumference norms in the Asian population (25). Individuals meeting three or more of the following criteria were diagnosed with MetS (4): triglycerides ≥150 mg/dL, fasting plasma glucose ≥100 mg/dL, HDL-C < 40 mg/dL (men) or <50 mg/dL (women), systolic blood pressure ≥130 mmHg or diastolic blood pressure ≥85 mmHg, and waist circumference ≥90 cm (men) or ≥80 cm (women).

**Figure 1:**
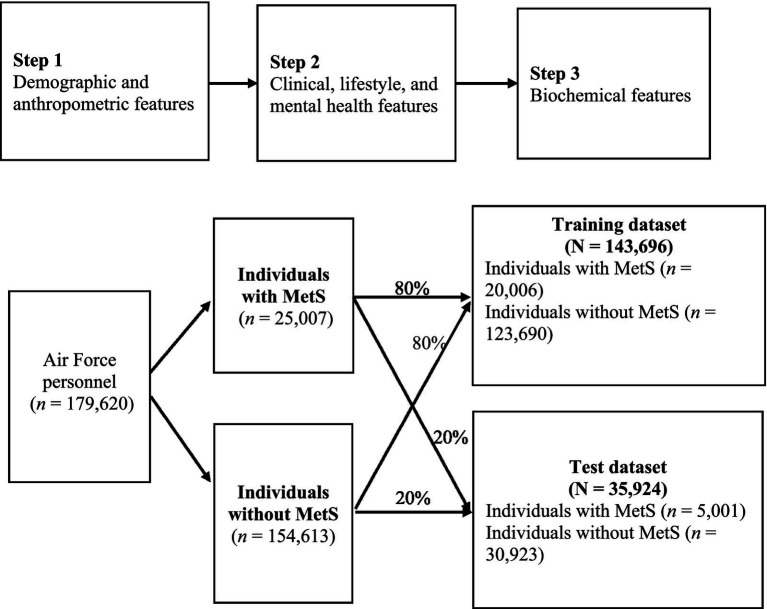
Process of data collection and machine learning. MetS, metabolic syndrome.

Because triglycerides, fasting plasma glucose, HDL-C, blood pressure, and waist circumference were used to define the MetS outcome, these variables were not included as predictors in the machine learning models to avoid circularity and redundancy. In addition, the BSRS total score overlapped with its five individual items, which were retained to provide more granular information. Therefore, seven features were excluded, and the remaining 29 features were retained for model development using the sequential three-step process described above (Figure 1).

### 2.4 Machine learning

A total of 29 key features of MetS were included in the models in three sequential steps (Figure 1). The contribution of each feature to model performance was evaluated. The dataset was divided into MetS and non-MetS groups at an 80:20 ratio, yielding training and test datasets of 143,696 and 35,924 Air Force personnel, respectively. To address class imbalance, the synthetic minority oversampling technique (SMOTE) was applied exclusively to the training dataset prior to model development, while the test dataset was left unchanged to ensure unbiased evaluation (26). For comparison, we also assessed two other approaches, adaptive synthetic sampling (ADASYN) and class weighting, both of which produced area under the receiver operating characteristic curve (AUC) values similar to those obtained with SMOTE (see Supplementary Table S1 for detailed results). Because SMOTE is well validated and widely applied in biomedical prediction research, it was selected as the primary method for handling class imbalance in this study.

Data preprocessing included standardization to ensure compatibility across models. Six machine learning algorithms—KNN, RF, LR, SVM, NN, and NB—were implemented via Python (version 3.8) with libraries including scikit-learn, imbalanced-learn, and SHapley Additive exPlanations (SHAP). Each model was initially trained with default hyperparameters, followed by optimization through grid search.

Hyperparameter optimization was conducted using grid search with 5-fold cross-validation for the RF, SVM, and NN models, whereas KNN, LR, and NB were implemented with standard or default configurations. For RF, the grid included variations in the number of trees, maximum depth, and minimum split size; for SVM, the penalty parameter (*C*) and kernel coefficient (*gamma*) were tuned with the RBF kernel; and for NN, hidden layer sizes, regularization (*alpha*), and learning rate were explored. The detailed parameter ranges for all models are provided in Supplementary Table S2.

Model performance was evaluated via multiple metrics, including accuracy, F1 score, precision, recall, specificity, and area under the receiver operating characteristic curve (AUC). Accuracy was calculated via the following equation: true positives (TP) + true negatives (TN) / (TP + false negatives [FN] + false positives [FP] + TN) = (TP + TN) / total sample count. The F1 score is the harmonic mean of precision and sensitivity. The precision was calculated as follows: TP / (TP + FP). Recall was calculated as follows: TP / (TP + FN). The specificity was calculated as follows: TN / (FP + TN).

The discriminatory ability of the models was visualized via receiver operating characteristic (ROC) curves. Feature importance was assessed via SHAP values, which provided insights into the top predictors for MetS in the male and female subgroups. These analyses identified the 10 most influential features for each subgroup, providing tailored insights into risk patterns.

### 2.5 Statistical analysis

Continuous variables are presented as means and standard deviations; categorical variables are shown as frequencies and percentages. Group comparisons between individuals with and without MetS were conducted using independent *t*-tests for continuous data and chi-square tests for categorical data. A *p*-value <0.05 indicated statistical significance.

## 3 Results

### 3.1 Cohort characteristics

The study included 179,620 active-duty Air Force personnel, of whom 83.5% were men. Table 1 summarizes the cohort characteristics. A total of 25,007 individuals (13.9%) met the criteria for MetS, whereas 154,613 (86.1%) did not. Individuals in the MetS group were significantly older than those in the non-MetS group (mean age: 34.36 ± 5.94 vs. 29.88 ± 6.79 years; *p* < 0.001). The proportion of men was also greater in the MetS group than in the non-MetS group (95.3% vs. 81.5%; *p* < 0.001). Mental health scores, excluding those for suicide attempts, were significantly higher in the MetS group. In addition, all biochemical measurements were significantly greater in the MetS group than in the non-MetS group.

**Table 1:** Demographic, anthropometric, disease, lifestyle, mental health, and biochemical features of the study cohort (*N* = 179,620).

Variable	Total	Metabolic syndrome				t/χ^2^	*p* value
		Yes (*n* = 25,007)		No (*n* = 154,613)			
	Mean (*SD*) or *n* (%)	Mean (SD)	*n* (%)	Mean (SD)	*n* (%)		
Demographic features							
Age	30.51 (6.85)	34.36 (5.94)		29.88 (6.79)		−108.40	<0.001
Sex						2947.08	<0.001
Women	29,720 (16.5)		1,178 (4.7)		28,542 (18.5)		
Men	149,900 (83.5)		23,829 (95.3)		126,071 (81.5)		
Anthropometric feature							
Body mass index	24.87 (3.79)	29.14 (3.35)		24.18 (3.39)		−216.78	<0.001
Clinical features							
Chronic disease						2198.08	<0.001
No	164,163 (91.4)		20,926 (83.7)		143,237 (92.6)		
Yes	15,457 (8.6)		4,081 (16.3)		11,376 (7.4)		
Family history						1766.32	<0.001
No	141,104 (78.6)		17,114 (68.4)		123,990 (80.2)		
Yes	38,516 (21.4)		7,893 (31.6)		30,623 (19.8)		
Lifestyle features							
Smoking						1743.10	<0.001
No	138,496 (77.1)		16,708 (66.8)		121,788 (78.8)		
Yes	41,124 (22.9)		8,299 (33.2)		32,825 (21.2)		
Betel nut chewing						1558.71	<0.001
No	171,078 (95.2)		22,585 (90.3)		148,493 (96.0)		
Yes	8,542 (4.8)		2,422 (9.7)		6,120 (4.0)		
Alcohol consumption						630.12	<0.001
No	166,262 (92.6)		22,181 (88.7)		144,081 (93.2)		
Yes	13,358 (7.4)		2,826 (11.3)		10,532 (6.8)		
Physical activity						274.18	<0.001
No	7,573 (4.2)		950 (3.8)		6,623 (4.3)		
Occasionally	50,196 (27.9)		7,971 (31.9)		42,225 (27.3)		
1 to 2 times a week	71,180 (39.6)		9,813 (39.2)		61,367 (39.7)		
3 to 4 times a week	50,671 (28.2)		6,273 (25.1)		44,398 (28.7)		
Rapid fatigue during exercise						2142.28	<0.001
No	164,477 (91.6)		21,012 (84.0)		143,465 (92.8)		
Yes	15,143 (8.4)		3,995 (16.0)		11,148 (7.2)		
Infection within 1 month						3.24	0.072
No	179,266 (99.8)		24,946 (99.8)		154,320 (99.8)		
Yes	354 (0.2)		61 (0.2)		293 (0.2)		
Regular medication use						142.20	<0.001
No	175,403 (97.7)		24,155 (96.6)		151,248 (97.8)		
Yes	4,217 (2.3)		852 (3.4)		3,365 (2.2)		
Mental health features							
Total BSRS score	0.94 (1.93)	1.16 (2.11)		0.91 (1.90)		−17.81	<0.001
BSRS-1 Sleep score	0.27 (0.55)	0.35 (0.61)		0.26 (0.54)		−20.45	<0.001
BSRS-2 Tension score	0.19 (0.45)	0.22 (0.48)		0.18 (0.44)		−11.03	<0.001
BSRS-3 Anger score	0.20 (0.48)	0.26 (0.54)		0.19 (0.47)		−17.83	<0.001
BSRS-4 Mood score	0.16 (0.43)	0.19 (0.47)		0.15 (0.42)		−13.28	<0.001
BSRS-5 Inferiority score	0.12 (0.39)	0.15 (0.42)		0.12 (0.38)		−9.31	<0.001
Suicide attempt score	0.02 (0.15)	0.02 (0.16)		0.02 (0.15)		−3.73	<0.001
Biochemical features							
Aspartate transaminase level	20.60 (12.92)	26.04 (16.23)		19.72 (12.07)		−59.00	<0.001
Alanine transaminase level	24.77 (21.91)	41.49 (30.58)		22.07 (18.82)		−97.50	<0.001
Blood urea nitrogen level	13.00 (3.10)	13.14 (3.28)		12.97 (3.06)		−7.69	<0.001
Creatinine level	0.90 (0.20)	0.94 (0.30)		0.89 (0.18)		−27.27	<0.001
Red blood cell count	5.15 (0.53)	5.35 (0.50)		5.12 (0.52)		−66.36	<0.001
White blood cell count	6.67 (1.67)	7.51 (1.82)		6.53 (1.60)		−79.81	<0.001
Hemoglobin level	15.00 (1.35)	15.55 (1.16)		14.91 (1.35)		−77.80	<0.001
Hematocrit level	44.67 (3.55)	45.99 (3.08)		44.46 (3.57)		−71.25	<0.001
Platelet count	261.24 (54.93)	271.87 (56.68)		259.52 (54.45)		−32.13	<0.001
Total cholesterol level	179.33 (33.80)	193.83 (36.06)		176.99 (32.83)		−69.34	<0.001
Uric acid level	6.24 (1.44)	7.13 (1.45)		6.10 (1.38)		−104.49	<0.001
Low-density lipoprotein cholesterol level	111.57 (30.64)	122.97 (31.92)		109.73 (30.03)		61.39	<0.001
MetS components							
Waist circumference						43082.98	<0.001
Normal	131,192 (73.0)		4,751 (19.0)		126,441 (81.8)		
Abnormal	48,428 (27.0)		20,256 (81.0)		28,172 (18.2)		
Triglyceride level						57528.45	<0.001
Normal	146,281 (81.4)		6,684 (26.7)		139,597 (90.3)		
Abnormal	33,339 (18.6)		18,323 (73.3)		15,016 (9.7)		
High-density lipoprotein cholesterol level						35089.92	<0.001
Normal	147,856 (82.3)		10,099 (40.4)		137,757 (89.1)		
Abnormal	31,764 (17.7)		14,908 (59.6)		16,856 (10.9)		
Blood pressure						30075.96	<0.001
Normal	127,733 (71.1)		6,251 (25.0)		121,482 (78.6)		
Abnormal	51,887 (28.9)		18,756 (75.0)		33,131 (21.4)		
Fasting plasma glucose level						27410.06	<0.001
Normal	149,090 (83.0)		11,633 (46.5)		137,457 (88.9)		
Abnormal	30,530 (17.0)		13,374 (53.5)		17,156 (11.1)		
Waist circumference (cm)	82.42 (10.44)	94.31 (8.24)		80.50 (9.44)		−240.82	<0.001
Triglyceride level (mg/dL)	108.13 (74.75)	205.95(107.13)		92.31 (53.27)		−164.49	<0.001
High-density lipoprotein cholesterol level (mg/dL)	51.31 (11.87)	40.57 (8.11)		53.05 (11.46)		211.68	<0.001
Systolic blood pressure	120.77 (12.66)	132.37 (11.78)		118.90 (11.76)		−167.82	<0.001
Diastolic blood pressure	73.46 (10.25)	82.47 (10.30)		72.00 (9.47)		−150.80	<0.001
Fasting plasma glucose	93.32 (12.32)	102.84 (22.20)		91.78 (8.93)		−77.77	<0.001

SD, standard deviation; BSRS, Brief Symptom Rating Scale.

The chi-square test revealed sex-specific patterns in the prevalence of MetS and its component abnormalities (Table 2). Among men, the most common abnormality was elevated blood pressure (32.9%), followed by increased waist circumference (28.4%), elevated triglycerides (21.3%), hyperglycemia (19.0%), and reduced HDL-C (17.6%). In women, increased waist circumference was most prevalent (19.9%), followed by reduced HDL-C (17.9%), elevated blood pressure (8.9%), hyperglycemia (7.0%), and elevated triglycerides (5.0%).

**Table 2:** Sex-specific prevalence of metabolic syndrome by its component abnormality of the cohort study (*N* = 179,620).

Metabolic components abnormality	Men			Women			*p*
	Total (*N* = 149,900) n (%)	MetS (*N* = 23,829) n (%)	Non-MetS (*N* = 126,071) n (%)	Total (*N* = 29,720) n (%)	MetS (*N* = 1,178) n (%)	Non-MetS (*N* = 28,542) n (%)	
Increased waist circumference	42,509 (28.4)	19,191 (80.5)	23,318 (18.5)	5,919 (19.9)	1,065 (90.4)	4,854 (17.0)	<0.001
	*p* < 0.001			*p* < 0.001			
Elevated triglycerides	31,854 (21.3)	17,698 (74.3)	14,156 (11.2)	1,485 (5.0)	625 (53.1)	860 (3.0)	<0.001
	*p* < 0.001			*p* < 0.001			
Reduced HDL-C	26,444 (17.6)	13,928 (58.4)	12,516 (9.9)	5,320 (17.9)	980 (83.2)	4,340 (15.2)	0.284
	*p* < 0.001			*p* < 0.001			
Elevated blood pressure	49,246 (32.9)	18,109 (76.0)	31,137 (24.7)	2,641 (8.9)	647 (54.9)	1,994 (7.0)	<0.001
	*p* < 0.001			*p* < 0.001			
Hyperglycemia	28,456 (19.0)	12,777 (53.6)	15,679 (12.4)	2,074 (7.0)	597 (50.7)	1,477 (5.2)	<0.001
	*p* < 0.001			*p* < 0.001			

MetS, metabolic syndrome; HDL-C, high density lipoprotein cholesterol.

When examining the prevalence of MetS within each abnormality, men with increased waist circumference (80.5%), elevated blood pressure (76.0%), or elevated triglycerides (74.3%) were most likely to meet MetS criteria, followed by reduced HDL-C (58.4%) and hyperglycemia (53.6%). In women, increased waist circumference (90.4%) and reduced HDL-C (83.2%) were the strongest correlates of MetS, followed by elevated blood pressure (54.9%), elevated triglycerides (53.1%), and hyperglycemia (50.7%).

Overall, 17.6% of men and 17.9% of women presented with reduced HDL-C (*p* = 0.284), indicating no significant sex difference in overall prevalence. However, when stratified by MetS status, reduced HDL-C was observed in 58.4% of men with MetS versus 9.9% without MetS, and in 83.2% of women with MetS versus 15.2% without MetS. These findings suggest that while the overall prevalence of reduced HDL-C was comparable between sexes, within the MetS subgroup, women were disproportionately more likely than men to exhibit reduced HDL-C.

### 3.2 Model performance

The six machine learning models demonstrated accuracies ranging from 0.80 to 0.89. Among them, the RF, LR, SVM, and NN models achieved the highest overall accuracy (0.89). The NN model had the highest F1 score (0.51), indicating balanced performance in precision and recall. The SVM model had the highest precision (0.73), followed by RF (0.68), LR (0.67), NN (0.65), KNN (0.57), and NB (0.37) models. The model recall values ranged from 0.31 to 0.56, and the specificity values ranged from 0.84 to 0.98 (Table 3).

**Table 3:** Model performance in predicting MetS.

Model	Accuracy	F1 score	Precision	Recall	Specificity	AUC
K-nearest neighbor	0.87	0.41	0.57	0.32	0.96	0.80
Random forest	0.89	0.47	0.68	0.36	0.97	0.89
Logistic regression	0.89	0.49	0.67	0.38	0.97	0.89
Support vector machine	0.89	0.44	0.73	0.31	0.98	0.84
Neural network	0.89	0.51	0.65	0.43	0.96	0.89
Naïve Bayes	0.80	0.44	0.37	0.56	0.84	0.81

MetS, metabolic syndrome; AUC, area under the receiver operating characteristic curve.

**Figure 2:**
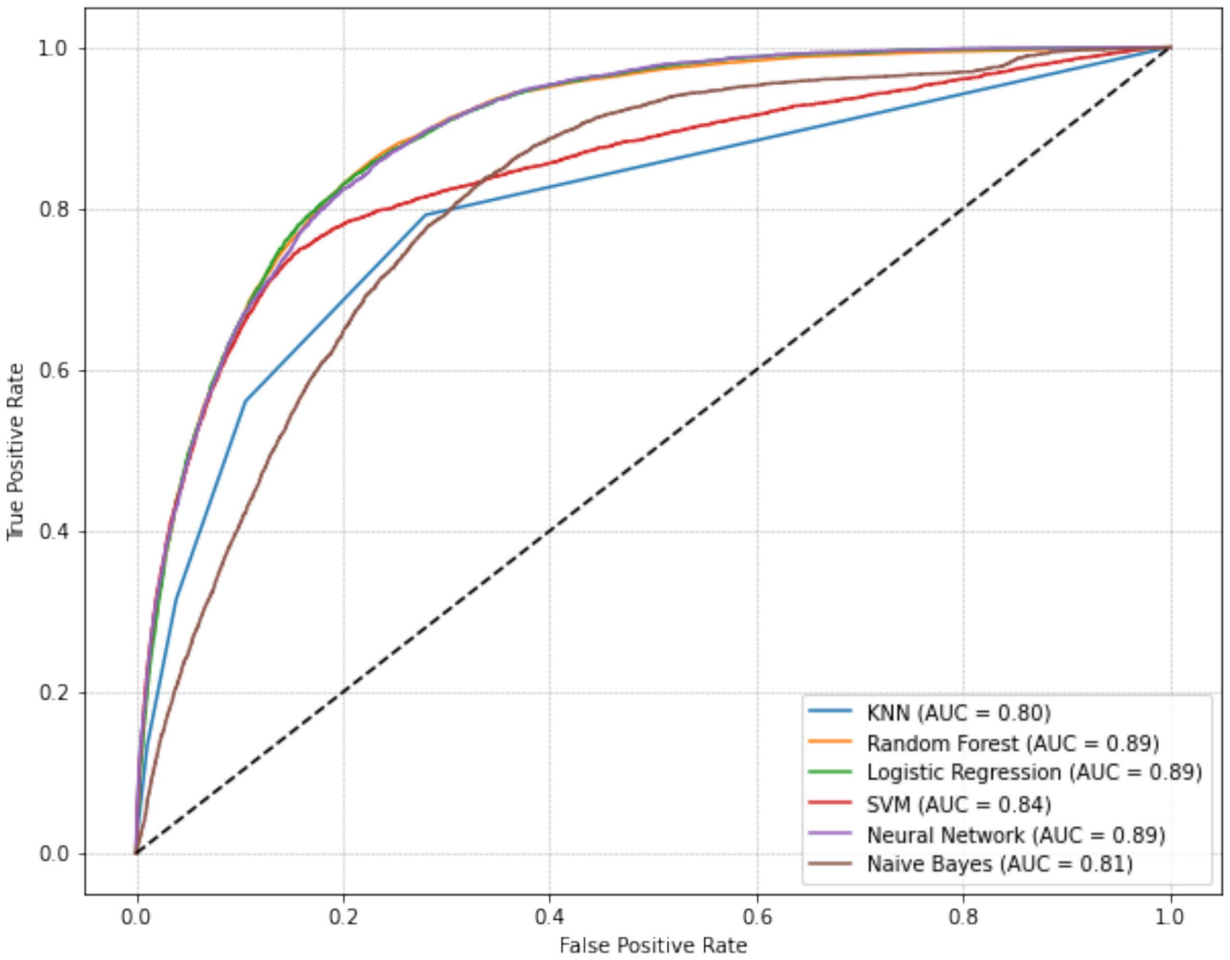
Receiver operating characteristic (ROC) curves of six machine learning models for predicting metabolic syndrome, with Logistic Regression achieving the highest AUC (0.894), followed by Random Forest, SVM, and Neural Network.

For predicting MetS events, the LR, RF, and NN models yielded the highest AUC values (all rounded to 0.89), followed by SVM (0.84), NB (0.81), and KNN (0.80; Figure 2). Pairwise comparisons using DeLong’s test indicated that LR achieved the highest AUC (0.894), significantly exceeding RF (0.890; ΔAUC = 0.004; p_FDR < 0.001) and NN (0.873; ΔAUC = 0.021; p_FDR < 0.001; Table 4). RF also significantly outperformed NN (ΔAUC = 0.017; p_FDR < 0.001). The overall performance ranking was LR > RF > SVM > NN, with all four significantly outperformed NN and NB (all p_FDR < 0.001). Although the absolute AUC differences among LR, RF, and NN were small (≤0.021), they remained statistically significant after multiple-comparison adjustment.

**Table 4:** Pairwise comparison of area under the receiver operating characteristic curve values among six machine learning models for predicting metabolic syndrome using DeLong’s test.

Model A	Model B	AUC A	AUC B	ΔAUC	Z	p_FDR_BH
Logistic Regression	Naive Bayes	0.894	0.818	0.076	38.268	0
KNN	Logistic Regression	0.818	0.894	−0.076	35.574	2.65E-276
Random Forest	Naive Bayes	0.890	0.818	0.071	35.505	2.06E-275
KNN	Random Forest	0.818	0.890	−0.072	34.004	7.27E-253
KNN	SVM	0.818	0.882	−0.064	30.554	1.52E-204
SVM	Naive Bayes	0.882	0.818	0.064	28.313	6.03E-176
KNN	Neural Network	0.818	0.873	−0.055	24.271	8.50E-130
Naive Bayes	Neural Network	0.818	0.873	−0.055	23.474	1.42E-121
Logistic Regression	Neural Network	0.894	0.873	0.021	16.416	2.44E-60
Random Forest	Neural Network	0.890	0.873	0.017	13.053	9.11E-39
Logistic Regression	SVM	0.894	0.882	0.012	11.970	6.99E-33
SVM	Neural Network	0.882	0.873	0.009	8.694	4.38E-18
Random Forest	SVM	0.890	0.882	0.007	7.515	6.58E-14
Random Forest	Logistic Regression	0.890	0.894	−0.004	4.890	1.08E-06
KNN	Naive Bayes	0.818	0.818	0.0002	0.059	0.953

KNN, k-nearest neighbors; SVM, support vector machine; AUC, area under the receiver operating characteristic curve; ΔAUC, difference in AUC between models; Z, test statistic; p_FDR_BH, *p*-value adjusted for multiple comparisons using the Benjamini–Hochberg false discovery rate method.

### 3.3 Sex-specific MetS predictors

**Figure 3:**
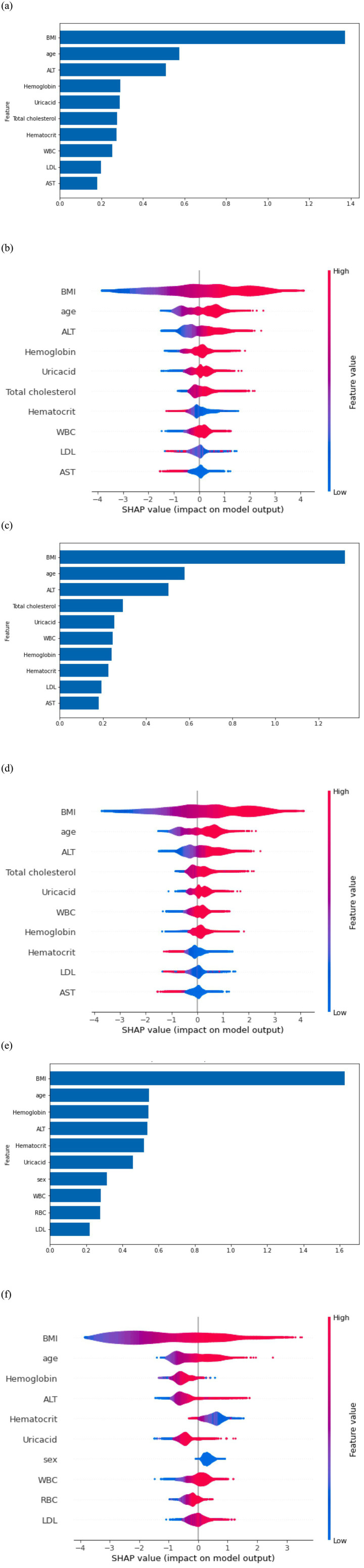
**(a)** Feature importance in the overall model. **(b)** Shapley Additive exPlanations (SHAP) on the overall model output. **(c)** Feature importance in the model for men. **(d)** SHAP on model output for men. **(e)** Feature importance in the model for women. **(f)** SHAP on model output for women.

All six models passed the Hosmer–Lemeshow test, indicating good model fit. The top 10 predictive features for MetS identified by SHAP analysis are shown in Figure 3. Across the entire cohort, BMI, age, ALT level, hemoglobin, and uric acid were the five most strongly predictive features (Figures 3a,b). Features related to clinical, lifestyle, and mental health contributed minimally to the prediction. For men, BMI, age, ALT level, total cholesterol, and uric acid level were the most influential predictors (Figures 3c,d). For women, BMI, age, hemoglobin, ALT level, and hematocrit were the most predictive features (Figures 3e,f). Across both sexes, BMI, age, and ALT levels were consistently identified as the most influential predictors.

## 4 Discussion

Maintaining optimal health in military personnel is crucial as poor health can hinder their ability to fully dedicate themselves to national security (3). This study represents a novel attempt to explore sex-based differences in early MetS detection among Air Force personnel via machine learning models. We systematically developed prediction models by sequentially incorporating demographic, anthropometric, clinical, lifestyle, mental health, and biochemical features. Six machine learning algorithms were employed, and the models were tailored by sex. Model performance and feature importance were subsequently evaluated.

Among the models, the LR model exhibited the most robust performance in predicting MetS, achieving an accuracy and AUC of 0.89. The RF model also performed well. The findings revealed that BMI, age, and ALT level were the three strongest predictors of MetS in Air Force personnel. For men, total cholesterol and uric acid were also key predictors, whereas hemoglobin and hematocrit were influential for women. These results emphasize the importance of tailoring early MetS risk detection models to account for sex-based differences.

Beyond model-derived predictors, our analysis of individual MetS components revealed a noteworthy sex-specific pattern in HDL-C abnormalities. Although the overall prevalence of reduced HDL-C was similar between men and women, within the MetS subgroup women were disproportionately more likely than men to exhibit reduced HDL-C. This observation aligns with findings from Ramezankhani et al., who reported that HDL-C decline was more strongly associated with MetS progression in women than in men (27). While HDL-C was not included as a predictor variable in our models due to its role in defining the MetS outcome, the sex-specific distribution observed in our study underscores the importance of developing tailored prediction models that reflect distinct risk profiles in men and women.

Elevated ALT levels were consistently identified as a significant predictor of MetS in both sexes. ALT is a specific marker of liver function, as it is predominantly localized in hepatocytes and is released into the bloodstream following liver cell damage (28). Its elevation is strongly linked to fatty liver disease, a condition that leads to lipid accumulation in the liver and other organs, ultimately promoting insulin resistance—a key factor in the development and progression of MetS (29). In contrast, AST reflects systemic enzymatic activity, as it is found in multiple tissues, including the heart, brain, and skeletal muscles, making it less specific to liver function. The liver-specific role of ALT in MetS development explains its stronger association with MetS than that of AST. Our findings align with those of previous studies conducted among military personnel, which reported the significance of ALT in MetS prediction (30). Additionally, elevated ALT levels have been associated with occupational stress and fatigue, which are factors prevalent among military personnel (31).

In our study, biochemical parameters exhibited greater specificity for MetS prediction than did disease-related and lifestyle factors. This may be attributed to the gradual emergence of biochemical abnormalities during the pathophysiological progression of MetS. Prioritizing these biochemical markers in screening processes can enhance early detection and improve intervention strategies for MetS. In contrast, disease-related and lifestyle factors, such as smoking and alcohol consumption, are well-established risks that indirectly contribute to biochemical changes leading to MetS (32). This highlights the need for an integrated approach that combines biochemical monitoring with lifestyle interventions to provide comprehensive strategies for preventing and managing MetS.

Interestingly, mental health indicators were not identified as key predictors of MetS in this cohort. One possible explanation is the unique resilience and coping mechanisms instilled by military training, which may mitigate the impact of psychological stress on metabolic health (33, 34). The strong emphasis on discipline, physical fitness, and mental toughness in military culture likely contributes to this resilience (35). However, two alternative explanations should also be considered. First, military recruitment and retention policies generally exclude individuals with severe mental health conditions, leading to a more homogeneous cohort with less variability in psychological indicators. Secondly, reliance on self-reported measures of psychological distress may limit the ability to fully capture real-world mental health conditions. In particular, social desirability bias may have led participants to underreport BSRS-5 symptoms, potentially attenuating the observed associations with MetS and highlighting the need for further research and targeted intervention strategies.

This study has several limitations. First, the lack of genetic and dietary data limits the comprehensiveness of prediction models. The incorporation of such data in future studies may increase model accuracy. Second, because the study was conducted on Air Force personnel, the generalizability of the findings to other populations remains uncertain. Finally, feature importance was analyzed via cross-sectional data, which hindered the investigation of causal relationships between the features and MetS. Large-scale longitudinal studies must be conducted in the future.

## 5 Conclusion

We developed sex-specific MetS prediction models for military personnel that incorporate demographic, anthropometric, clinical, lifestyle, mental, and biochemical features. The LR model achieved the highest accuracy and AUC for MetS prediction, followed closely by the RF model. BMI, age, and ALT level emerged as the most important predictors of MetS in both sexes. For men, total cholesterol and uric acid were also significant, whereas hemoglobin and hematocrit were influential for women. These findings highlight the importance of sex-based differences in early MetS risk detection and the utility of early prediction models in routine health screenings.

Based on these results, population-level interventions should emphasize structured weight management, education on liver health (e.g., reducing alcohol consumption and unhealthy diets), and age-specific health screenings for all personnel. For men, targeted strategies should include dietary modifications to lower total cholesterol and uric acid levels, combined with regular monitoring to enable early management of these risks. For women, interventions should prioritize nutritional support to maintain adequate hemoglobin and hematocrit levels, along with routine screening to detect and address underlying causes of deficiencies. Tailoring preventive strategies to sex-specific risk profiles may enhance early detection and optimize the management of MetS in military populations.

## Data Availability

The raw data supporting the conclusions of this article will be made available by the authors, without undue reservation.
