# Supplementary material for: Early risk detection of metabolic syndrome using sex-specific machine learning models in military personnel
Source: Front Public Health. 2025 Oct 31;13:1625461. doi: 10.3389/fpubh.2025.1625461 (PMC12617432; doi:10.3389/fpubh.2025.1625461)
Supplement: Supplementary file 1 [file Data_Sheet_1.PDF]

Supplementary Table S1. Comparison of model performance using different imbalance-handling techniques across six machine learning algorithms.

Model	AUC_SMOTE	AUC_ADASYN	AUC_ class weights
K-nearest neighbor	0.818	0.813	0.805
Random forest	0.890	0.887	0.892
Logistic regression	0.894	0.894	0.895
Support vector machine	0.882	0.878	0.891
Naïve Bayes	0.818	0.818	0.812
Neural network	0.873	0.865	0.898

Abbreviations: AUC = Area under the receiver operating characteristic curve; SMOTE = synthetic minority over-sampling technique; ADASYN = adaptive synthetic sampling.

Supplementary Table S2. Hyperparameter settings for machine learning models

Model	Hyperparameter Settings
RF	n_estimators = {100, 200, 500}; max_depth = {None, 10, 20}; min_samples_split = {2, 5}
SVM	C = {0.1, 1, 10}; gamma = {scale, 0.01, 0.001}; kernel = RBF
NN	hidden_layer_sizes = {(50,), (100,), (100, 50)}; alpha = {0.0001, 0.001}; learning_rate_init = {0.001, 0.01}
KNN	n_neighbors = 5; distance metric = Euclidean
LR	solver = lbfgs; max_iter = 1000; regularization parameter C = 1
NB	Gaussian distributional assumption; default variance smoothing

Abbreviation: RF = random forest; SVM = support vector machine; NN = neural network; KNN = K-nearest neighbor; LR = logistic regression; NN = naïve Bayes.
